# Genetic Disorders Underlying Polyhydramnios and Congenital Hypotonia: Three Case Reports and a Review of the Literature

**DOI:** 10.7759/cureus.50331

**Published:** 2023-12-11

**Authors:** Niki Dermitzaki, Themistoklis Loukopoulos, Athanasios Zikopoulos, Anastasia Vatopoulou, Sofoklis Stavros, Chara Skentou

**Affiliations:** 1 Neonatal Intensive Care Unit, University Hospital of Ioannina, Ioannina, GRC; 2 Medicine, University of Ioannina, Ioannina, GRC; 3 Obstetrics and Gynecology, University Hospital of Ioannina, Ioannina, GRC; 4 Obstetrics and Gynecology, Royal Cornwall Hospital, Cornwall, GBR; 5 Obstetrics and Gynecology, University Hospital Attikon, National and Kapodistrian University of Athens, Athens, GRC; 6 Obstetrics and Gynecology, University of Ioannina, Ioannina, GRC

**Keywords:** fetal medicine, neaonatal neurological disorders, obstetric counseling, prenatal diagnosis, genetic disorders, polyhydramnios

## Abstract

An abnormal rise in the amount of amniotic fluid is a frequent prenatal observation called polyhydramnios, which can indicate a number of underlying problems. Even while it frequently goes undiagnosed during pregnancy, it may be linked to dangerous fetal illnesses. In three cases of newborns with congenital hypotonia, polyhydramnios was the sole prenatal symptom reported in this study. This fact highlights the significance of understanding the possible connection between genetic abnormalities or neurological problems and polyhydramnios, underscoring the responsibility obstetricians have in educating expectant mothers who are at potential risk for these uncommon but serious illnesses. Whole-genome sequencing (WES), an advanced kind of prenatal testing, is essential for determining genetic reasons and assisting families in making decisions. Working together with specialists in fetal medicine is crucial in guaranteeing the best possible treatment and results for the mother and child.

## Introduction

Polyhydramnios refers to an abnormal increase in the volume of amniotic fluid. It is observed in 1-2% of singleton pregnancies and is more commonly diagnosed by fetal ultrasound in the second or third trimester. Quantitative sonographic criteria include amniotic fluid index (AFI) >24cm or a single deepest pocket (SDP) >8cm [1]. The most common causative mechanisms include decreased fetal swallowing or increased fetal urine output. Diabetes is the most common maternal contributing factor to polyhydramnios. Fetal conditions associated with polyhydramnios include gastrointestinal tract obstruction, chromosomal abnormalities such as Down syndrome, structural central nervous system anomalies, compressive airway lesions, neurological and neuromuscular disorders, congenital infections and pathologies leading to non-immune hydrops fetalis, placental tumors, and twin to twin transfusion syndrome in a non-diagnosed monochorionic twin pregnancy. In up to 60% of cases, no predisposing condition is identified antenatally, and the polyhydramnios is considered idiopathic. However, in up to a quarter of cases of presumed idiopathic polyhydramnios, an abnormality is identified postnatally, including structural malformations, genetic syndromes, and neurological disorders [2]. Disorders associated with congenital hypotonia often remain undiagnosed prenatally due to the late presentation of subtle sonographic signs [3]. Fetal neurological dysfunction has been associated with polyhydramnios, and the causative mechanism has been hypothesized to be swallowing impairment [4]. Here, we present three cases of neonates with congenital hypotonia, the only manifestation of which, antenatally, was increased amniotic fluid volume.

## Case presentation

Case 1

The first case involves a male neonate born to a 38-year-old gravida 3 para 3 (G3P3) mother. The woman was diagnosed with gestational diabetes mellitus -controlled by diet- in the second trimester, a major risk factor for polyhydramnios. No other complications were observed during the pregnancy. However, polyhydramnios was detected at 34 weeks of gestation with the absence of obvious fetal structural abnormalities. At the same ultrasound session, fetal growth restriction was diagnosed since the estimated fetal weight (EFW) was in the seventh percentile for gestational age (Table 1).

**Table 1:** Ultrasound scan at 34 weeks revealed a small for gestational age fetus BPD - biparietal diameter (the diameter between the two sides of the head; OFD - occipital-frontal diameter; HC - head circumference; TCD - transverse cerebellar diameter; TAD: transverse abdominal diameter; APAD - anterior-posterior abdominal diameter; AC - abdominal circumference; HL - humeral length; FL - femur length

Fetal anatomy at 34 weeks of gestation	
BPD	79.6 mm
OFD	107.4 mm
HC	293.7 mm
TCD	45.1 mm
TAD	82.0 mm
APAD	83.0 mm
AC	259.2 mm
HL	58.7 mm
FL	65.2 mm
BPD/ OFD	0.74
HC/AC	1.13
BPD / FL	1.22
Estimation of fetal weight	1.797 g
(BPD-HC-AC-FL)	
Percentile	5.5

A male neonate was delivered vaginally at 40 weeks of gestation with a low birth weight of 2260 grams. He was admitted to the neonatal intensive care unit (NICU) due to severe hypotonia. Physical examination at birth revealed severe truncal hypotonia, depressed neonatal reflexes, and hyperactive deep tendon reflexes. He had no dysmorphic features except for mild micrognathia. Brain magnetic resonance imaging (MRI) showed no structural abnormalities but did reveal increased signal intensity of the basal ganglia and the thalami on T1 weighted images and elevation of choline on magnetic resonance spectroscopy. Genetic testing was performed using whole genome sequencing (WGS), and heterozygous missense variant c.2375A>G in the methyl-binding domain 5 (MBD5) gene was detected.

On admission to the NICU, he was managed conservatively with nasogastric tube feeding and respiratory support with non-invasive ventilation. Although weaning from a high-flow nasal cannula (HFNC) was achieved on the 19th day of life, no clinical improvement in hypotonia was noted. During his hospitalization, physiotherapy and a speech therapy feeding program were applied. He had a gastrostomy at three months. He was discharged at four months of age.

Case 2

**Figure 1:**
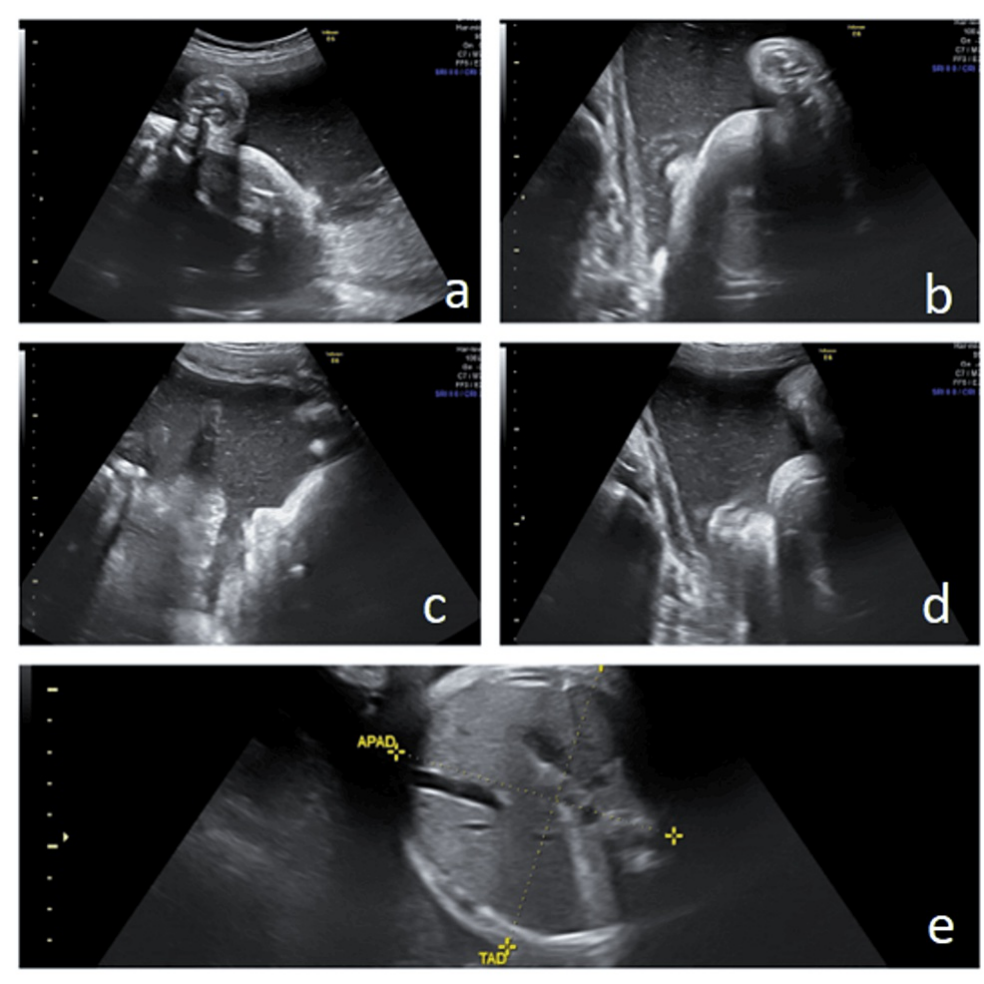
Ultrasound scan at 35 weeks demonstrates increased amniotic fluid index. The dimensions of abdomen are normal and the stomach is visible. During the scan, normal swallowing movements of the fetus were detected. a) First quadrant, b) second quadrant, c) third quadrant, d) fourth quadrant, e) fetal abdomen with normal size for the gestational age

The second case presented involves a male neonate born to a 19-year-old (G0P0) mother. Fetal ultrasound at 35 weeks of gestation revealed polyhydramnios. A fetal anatomic survey detected no obvious abnormalities, and the mother was not diagnosed with diabetes (Figure 1). The neonate was born at 36 weeks gestation, weighing 2410 grams by emergency cesarean section due to a non-reassuring non-stress test (NST). He was transferred to the NICU due to hypotonia. Upon admission, severe hypotonia and absent neonatal reflexes were noted. He did not exhibit any dysmorphic phenotypic features. A brain MRI was performed on the second day of life, revealing abnormal signal intensity in the globus pallidus and thalami. No malformations of the central nervous system were detected. During his hospitalization, serial cranial ultrasound scans were performed and showed increased echogenicity of the basal ganglia and the thalami. Whole exome sequencing (WES) revealed heterozygous variant c.388G>A in the sarcoglycan epsilon (SGCE) gene.

The neonate was managed with non-invasive ventilation and nasogastric tube feeding. Gradually, he developed lower limb hypertonia, hyperactive deep tendon reflexes, and clonus. A multidisciplinary approach was applied, including physiotherapy and a speech therapy feeding program. The infant was gradually weaned from non-invasive ventilation by the age of five months. However, no improvement was noted concerning oral feeding. He underwent a gastrostomy at the age of six months and was discharged from the hospital at the age of 12 months.

Case 3

**Figure 2:**
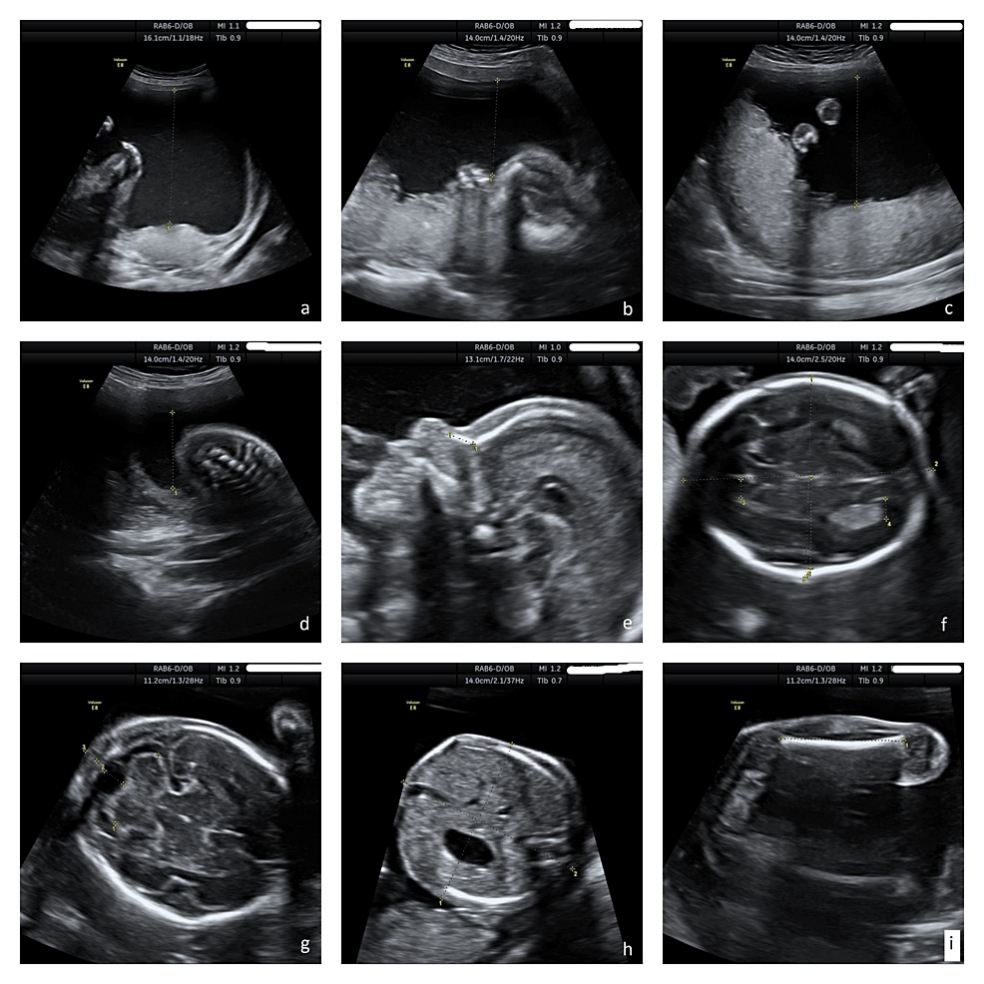
Ultrasound scan at 21 weeks demonstrates increased amniotic fluid index and a large for gestational age fetus, without any major abnormality a) First quadrant, b) second quadrant, c) third quadrant, d) fourth quadrant, e) nasal bone, f) biparietal diameter, g) occipital-frontal diameter, h) abdominal dimensions, i) femur length

The third case refers to a male preterm neonate born to a 40-year-old G1P1 mother. The fetus was noted to be large for gestational age (LGA) from the 21st week of gestation. Amniocentesis was performed, and the fetal karyotype, assessed by array comparative genomic hybridization (CGH), was normal. Subsequently, polyhydramnios was detected at 32 weeks of gestation (Figure 2). The neonate was born at 34 weeks of gestation, with a birth weight of 4680 g, by an emergency cesarean section due to non-reassuring NST. He was admitted to the NICU due to respiratory distress and hypotonia. On physical examination at birth, in addition to generalized hypotonia, macrosomia, macrocephaly, and dysmorphic facial characteristics were noted, including frontal bossing, broad nasal root, and low-set dysmorphic ears. Echocardiography showed a large atrial septal defect. Cranial ultrasound at birth revealed bilateral ventricular dilatation. Serial cranial ultrasound scans and MRI of the brain were performed during his hospitalization, and progressively worsening hydrocephalus ex-vacuo was noted. Genetic testing using WES was performed, and a pathogenic variant in exon 6 of the PIK3CA gene was identified.

After admission to the NICU, he required respiratory support, initially with mechanical ventilation and subsequently with non-invasive ventilation until the age of five months. Due to his inability to feed orally, a nasogastric catheter was used for feeding. There was no improvement regarding hypotonia of the infant and he was discharged at the age of seven months.

## Discussion

Polyhydramnios is one of the most frequent ultrasound antenatal findings. The diagnosis of polyhydramnios should be followed by a detailed anatomic survey of the fetus to exclude fetal malformation [2]. Pregnancies diagnosed with polyhydramnios in the third trimester are at significantly increased risk for chromosomal anomalies compared to women with normal amniotic fluid volume (4.1% vs. 0.12%, respectively) [5]. Neurologic disorders and developmental delay are also significantly more prevalent in school-aged children from pregnancies complicated by polyhydramnios compared to controls (9.7% vs. 3%, respectively) [4]. However, in many cases, polyhydramnios is considered idiopathic by exclusion as no underlying cause can be identified antenatally. According to published studies, in 10-28% of the cases of unexplained hydramnios, abnormalities in the infant are identified postnatally, during the neonatal period, or even later at follow-up [6-8]. Neonatal neurological and neuromuscular disorders are often associated with polyhydramnios and are usually diagnosed postnatally because of the late presentation and non-specificity of the sonographic findings [3].

We report three cases of neonates with congenital hypotonia identified postnatally, whose only prenatal manifestation was polyhydramnios. In a recent retrospective study, the reported prenatal detection rate of congenital hypotonia was 38.5% [3]. In the aforementioned study, polyhydramnios was detected in significantly more singleton pregnancies in the group of hypotonic neonates compared to the group of unaffected newborns (64% vs 3%, respectively). The authors proposed that targeted scanning combined with genetic testing in pregnancies demonstrating non-specific sonographic signs, such as hydramnios, persistent breech presentation, fetal growth restriction, and maternal reporting of reduced fetal movement would increase the detection rate of congenital hypotonia up to 88.5% [3].

Congenital hypotonia is a relatively rare condition. It varies in severity and has a wide range of underlying causes. Molecular genetic testing is often required to establish a definitive diagnosis. Of the available techniques, WES has the highest diagnostic utility [3]. AlBanji et al. reported that in 59% of cases of congenital hypotonia, WES established the final diagnosis. Prenatal WES could be performed in pregnancies with a high index of suspicion for a genetic neurological or neuromuscular disorder [9]. In all of the three cases described above, genetic testing using WES detected variants of uncertain significance in genes associated with neurodevelopmental disorders.

Genetic testing of the first infant revealed heterozygous missense variants in the MBD5 gene. MBD5 has transcriptional regulating functions, which are required for the proper functioning of the neurons. MBD5-associated neurodevelopmental disorder (MAND) includes a group of disorders characterized by disrupted MBD5 function with consequent neurodevelopmental impairment. Global developmental delays are consistently observed, including cognitive impairment, fine and gross motor delay, and speech impairment. In infants, hypotonia and feeding difficulties are quite common, as was the case with the neonate reported here [10].

In the second case described, WES identified a heterozygous variant in the SGCE gene. Although the detected missense variant is of uncertain clinical significance, mutations in SGCE are known to be the main genetic cause of inherited myoclonus dystonia, a syndrome characterized by myoclonus and dystonia of the upper part of the body. Epsilon-sarcoglycan protein is expressed in various tissues and is probably implicated in the development of organs, including the brain [11].

In the third case presented, WES revealed a pathogenic variant of unknown clinical significance in the PIK3CA gene. Somatic pathogenic variants of the PIK3CA gene have been identified in a group of heterogeneous disorders characterized by overgrowth phenotypes, referred to as PIK3CA-related overgrowth spectrum (PROS) [12]. Although this patient did not fulfill the clinical criteria for megalencephaly-capillary malformation-polymicrogyria syndrome (MCAP), an overgrowth syndrome included in PROS, he demonstrated macrocephaly, ventriculomegaly, and severe hypotonia, features described in the above syndrome [13].

## Conclusions

In summary, this case report of three neonates with polyhydramnios emphasizes the importance of recognizing the potential association of this condition with genetic syndromes and neurological disorders. This study highlights the vital role obstetricians play in counseling pregnant women with polyhydramnios about these rare but serious conditions.

It is imperative that healthcare providers engage in a thorough prenatal consultation, which includes a complete overview of the various causes of polyhydramnios, with particular attention to associated genetic syndromes. Because of the devastating impact these conditions can have on families, prenatal tests, such as whole-genome sequencing (WES), should be offered to determine the karyotype of the fetus. Therefore, fostering collaboration with fetal medicine experts is key to ensuring pregnant women receive the best possible care and make informed decisions, which lead to better outcomes for mother and child. 
